# Investigating colistin drug resistance: The role of high-throughput sequencing and bioinformatics

**DOI:** 10.12688/f1000research.18081.2

**Published:** 2019-05-20

**Authors:** Dickson Aruhomukama, Ivan Sserwadda, Gerald Mboowa

**Affiliations:** 1 Department of Medical Microbiology, College of Health Sciences, School of Biomedical Sciences, Makerere University, Kampala, 7072, Uganda; 2 Department of Immunology and Molecular Biology, College of Health Sciences, School of Biomedical Sciences, Makerere University, Kampala, 7072, Uganda

**Keywords:** Antibiotic resistance, Colistin resistance, Pan-drug resistance, Gram negative bacteria, Genomics, Bioinformatics

## Abstract

Bacterial infections involving antibiotic-resistant gram-negative bacteria continue to increase and represent a major global public health concern. Resistance to antibiotics in these bacteria is mediated by chromosomal and/or acquired resistance mechanisms, these give rise to multi-drug resistant (MDR), extensive-drug resistant (XDR) or pan-drug resistant (PDR) bacterial strains. Most recently, plasmid-mediated resistance to colistin, an antibiotic that had been set apart as the last resort antibiotic in the treatment of infections involving MDR, XDR and PDR gram-negative bacteria has been reported. Plasmid-mediated colistin resistant gram-negative bacteria have been described to be PDR, implying a state devoid of alternative antibiotic therapeutic options. This review concisely describes the evolution of antibiotic resistance to plasmid-mediated colistin resistance and discusses the potential role of high-throughput sequencing technologies, genomics, and bioinformatics towards improving antibiotic resistance surveillance, the search for novel drug targets and precision antibiotic therapy focused at combating colistin resistance, and antibiotic resistance as a whole.

## Introduction

In the recent past, old antibiotic classes previously deemed unfit for treatment of bacterial infections due to associated toxicity concerns have been recommended for the treatment of the same infections ^1, 2^. This has been attributed to the emergence of resistance to the most recently considered last line antibiotics, the carbapenems ^1, 2^. Carbapenem resistance has been documented in bacteria belonging to the Enterobacteriaceae family, *Acinetobacter baumannii* and *Pseudomonas aeruginosa* ^1, 2^. The adoption of the old antibiotic agent category in routine empirical treatment has witnessed the use of a number of antibiotics such as colistin ^1, 2^.

Despite this reversion, gram-negative bacteria continue to undergo chromosomal mutations, which render their respective treatments virtually impossible and hence a major threat to global public health. The effects of these antibiotic resistance mutations are further exacerbated by horizontal transfer of antibiotic resistance genes in the same bacteria. As such, this paper explores the current documented trends of colistin resistance in several African settings. Additionally, it also describes the evolution of antibiotic resistance to plasmid-mediated colistin resistance and the potential role of genomics and bioinformatics in precision antibiotic therapy targeted towards combating colistin resistance and antibiotic resistance.

## Colistin resistance trends in Africa

**Figure 1.**
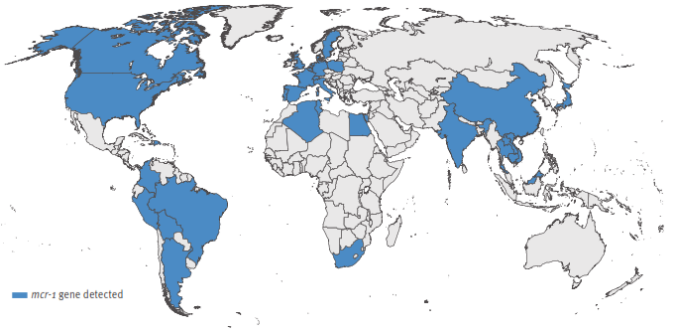
Data collected from 30 countries acknowledging the existence of the colistin resistant mcr-1 gene isolated from humans, the environment and animals. Reproduced from Xavier *et al.* ^4^ under a CC-BY 4.0 license.

Data on the antimicrobial resistance burden, particularly colistin resistance, in Africa remains limited ^3^. In 2014, the World Health Organization (WHO) reported that antimicrobial resistance surveillance in Africa was a particularly difficult feat due to the scarcity of viable medical data, statistical information and unreliable laboratory capacity ^3^. Despite this, African countries remain un-exempted from this worldwide antibiotic resistance trend that has emerged not only within hospital settings but also disseminated within communities. The available literature from African settings (i.e. South Africa, Algeria, Tunisia, and Egypt) has reported colistin resistance ( Figure 1) ^4– 7^. Beyond these settings, colistin resistance has also been reported in Uganda and Rwanda ^8– 10^.

## Evolution to plasmid-mediated colistin resistance in gram-negative bacteria

Colistin (polymyxin E) is part of an old generation of antibiotics ^11^ that form a family of cationic polypeptides. These are characterised by having a lipophilic fatty acyl side chain ^12– 14^. No exact mechanism of bacterial killing has been documented for polymyxins, especially in *Acinetobacter* spp ^14, 15^. However, a two-step mechanism has been described to elucidate their possible mechanism of action ^13, 14^.

The two-step mechanism involves i) initial binding to and permeabilization of the outer membrane and ii) the destabilization of the cytoplasmic membrane of the bacteria ^12– 14^. As a consequence, colistin functions by intercalating into the inner membrane following diffusion from the outer membrane across the periplasm and consequently causing the formation of pores, a phenomenon that results in bacterial lysis, which follows initial binding to bacterial surfaces ^12– 14^. Initial binding of colistin to the bacterial surface chiefly depends on the electrostatic interaction between the positively-charged colistin and the negatively charged phosphate group of lipid A, an endotoxic component on the lipopolysaccharide localised on the outer leaflet of the bacterial outer membrane ^12, 14^. 

The modifications of the lipid A which reduce and/or abolish the initial charge-based interaction of the polymyxins withbacteria and also the addition of either/or the 4-amino-4-deoxy-L-arabinose (L-Ara4N) and the phosphoethanolamine (PEtn) that ultimately form the basis of colistin resistance in bacteria are mediated by chromosomally encoded genes ^13– 16^. These genes are involved in the modulation of two-component regulatory systems; PmrA/PmrB and PhoP/PhoQ and *mgrB*, a negative regulator of the PhoP/PhoQ signaling system ^13– 16^ *.*

Although initially thought that this resistance could not be spread from cell to cell (plasmid-mediated) ^16^, currently studies have shown otherwise. These have alluded transfer of colistin resistance among bacteria via plasmids in horizontal gene transfer ^4, 16, 17^.

## Pan-drug resistance and characteristics of colistin-resistant gram-negative bacteria

The treatment of infections involving antibiotic-resistant gram-negative bacteria has become increasingly difficult overtime, a factor that has greatly contributed to high morbidity, mortality and high costs of health care ^18, 19^.

Currently, antibiotic resistance in these bacteria spans across several classes but likely follows a precise hierarchy of acquisition; this is mostly characterized by acquisition of “enhanced resistance” against more potent antibiotics following primary acquisition of “weaker resistance” against the less potent antibiotics alongside intrinsic resistance mechanisms in these bacteria, a trend that follows a Darwin’s like fashion ^20– 23^. These changes are a function of horizontal gene transfer, via conjugation, transformation and transduction ^24– 27^. 

Resistance in gram-negative bacteria has been seen to transit from being mediated by the extended spectrum β-lactamases, a group of enzymes that can be disseminated among bacteria ^28, 29^; these chiefly confer resistance against broad-spectrum cephalosporins. However, they also confer resistance to penicillins, monobactams and some carbapenems, particularly the *Klebsiella pnemoniae* carbapenemase, KPC ^28, 30, 31^. In the same hierarchy are AmpC β-lactamases that form another group of β-lactamases, derived from older broad spectrum β-lactamases. These provide an even more extended activity that includes resistance against the cephamycins alongside resistance to penicillins, monobactams and cephalosporins ^32– 34^. These enzymes have in recent times been shown to not only be limited to being encoded on the chromosomes of bacteria, but have also been documented to have the potential of being disseminated via plasmids in horizontal gene transfer ^28, 34, 35^ and also to co-exist with the extended spectrum β-lactamases ^36, 37^; factors that have made these bacteria “better resistant” to antibiotics. Next in the hierarchy are the carbapenemases, these enzymes are chiefly acquired in horizontal gene transfer and confer resistance to carbapenems alongside resistance to penicillins, broad-spectrum cephalosporins including cefepime, a fourth-generation cephalosporin, monobactams, aminoglycosides, quinolones and fluoroquinolones ^28, 38^. The development of resistance mediated by these enzymes to the different classes of antibiotics in these bacteria has been attributed to various factors among which is their use in therapy. This has not only abetted maintenance of resistance via selecting for resistance to these antibiotics in these bacteria but has also created a gap, a need for alternative antibiotics in therapy to replace the penicillins, β-lactams, carbapenems and the other classes of antibiotics used in the treatment of infections involving the drug-resistant gram-negative bacteria ^4, 16^.

Colistin, a polypeptide antibiotic, a relatively old antibiotic, has been currently relied upon to provide the ultimate line of refuge against infections caused by antibiotic-resistant gram-negative bacteria despite its previously documented impacts on health ^4, 16^. Colistin also appears to offer a choice in the face of almost no new antibiotics in production pipelines ^4, 16, 17^.

Worryingly, the use of colistin is under threat due to the emergence of plasmid-mediated colistin resistance involving *mcr* gene families ^4, 16^. This provides a new challenge as bacteria that express these resistance genes assume the lead in the antibiotic resistance hierarchy and are distinctively XDR or worse PDR ^16, 39– 42^.

Molecular studies previously done have reported colistin resistant gram-negative bacteria to also be resistant to an array of antibiotics ^43, 44^. These bacteria have also been reported to carry plasmids that have been found to carry alongside colistin resistance genes, β-lactamases ^43, 44^, carbapenemase encoding genes ^45^ and genes that code for resistances to other antibiotic classes that may include quinolones, fluoroquinolones and aminoglycosides ^13^. Additionally, the carriage of *mcr*-1 has been documented as a possible indicator of resistance to the third generation cephalosporins and carbapenems ^38, 44^. Furthermore, these genes have been found to be co–carried with other resistance determinants in plasmids ^13, 44, 46, 47^; these genes represent a novel mechanism of antibiotic resistance in bacteria and a threat to the existing antibiotic therapy. Worsening the situation is the ability of selection for colistin resistance via the use of the extended-spectrum cephalosporins. Additionally, the use of tetracycline and sulphonamides has also been reported to contribute to the dissemination of colistin mobile gene carrying plasmids ^44, 46^. Also, worth noting is plasmids that carry colistin resistance genes have also been found to mostly carry other antibiotic resistant genes ^13, 44– 46^.

## The role of high-throughput sequencing technologies and bioinformatics

Advances in technology including the rapidly growing field of genomics, are transforming clinical medicine ^48^ and high-throughput sequencing technology (HTS) is increasingly being used in clinical microbiology ^49^. HTS, with relatively simple benchtop technology and efficient genomic library preparation protocols, has significantly improved the capacity to perform low-cost, efficient whole-genome sequencing (WGS), and has made it a feasible tool to enhance clinical diagnostic investigations in near real-time ^48^. The processes generally involve culture-free parallel sequencing, producing vast quantities of genomic data that require modern computation techniques to assemble the genomic sequence reads as well as performing ensuing analyses that range from identifying the bacterial species or strain, antibiotic resistance mutations in the bacterial genomes, while ensuring the highest possible discriminatory power ever achieved by any technology ^49^. Apart from this, WGS of bacteria can identify genes associated with virulence and pathogenicity as well as discover new genetic mechanisms for virulence, pathogenicity and antibiotic resistance ^48, 50, 51^.

The identification and prediction of antibiotic-resistant microorganisms in clinical specimens solely by molecular means in the diagnostic microbiology laboratory is not novel ^52^. HTS technologies and computational tools offer unprecedented ability to sequence multitudes of bacterial genomes and enable interpretation of the resultant sequence information in near “real-time” ^52^.

WGS represents the pinnacle for bacterial strain characterization and epidemiological analyses. It is rapidly replacing traditional typing methods, antibiotic resistance gene detection and other molecular-based investigations in the near future. HTS technologies are rapidly evolving and their implementation in clinical and public health microbiology laboratories is increasing at a similar pace. These require standardized sample quality control, data interpretation, bioinformatics expertise, and infrastructure. The term ‘bioinformatics’ encompasses the handling and analysis of genomic sequence data, usually with the assistance of computer-based algorithms. Both ‘open source’ and commercially available bioinformatics programs/tools have been specifically developed for use in a clinical setting. However, many of practicing healthcare workers in current practice have limited bioinformatics knowledge ^48^.

Furthermore, genome plasticity, as well as pan-genomes that have the ability to influence bacterial resistome can only effectively be investigated using HTS and bioinformatics analyses ^48^. Understanding the bacterial genome dynamics is an important step in identifying the forces behind phenotypicantibiotic resistance and therefore allows for effective management of the antibiotic-resistant infections ^48^.

HTS and bioinformatics analyses have made it possible to employ comprehensive WGS-based surveillance of colistin resistance and antimicrobial resistance as a whole by enabling the rapid detection of colistin resistance (i.e. both chromosomal and plasmid-mediated) as well as resistance to other antibiotics; these have also made it possible to map how resistance spreads in a One-Health perspective in a way that was not possible before ^53, 54^. Furthermore, these have also allowed for rapid re-analysis of large datasets in silico, this has enabled the early detection as well as risk assessment particularly when new genes emerge ^53, 54^.

## The future direction of HTS

Antibiotic resistance in bacteria is generally a natural phenomenon ^55– 57^ though augmented by human behavior. Therefore, it is imperative to harness the best HTS technologies that sequence DNA at unprecedented speed, to enable previously unimaginable scientific achievements and novel biological applications ^53^. Such applications of genomics tools have revolutionized microbial ecological studies and drastically expanded our view on the previously underappreciated microbial world ^54^ including acquisition and transmission dynamics of antibiotic resistance. Single-Molecule Real-Time (SMRT) sequencing (Pacific Biosciences Inc.) in clinical microbiology has finally been realized at many levels in health care systems in the developing world and relatively only used during isolated scenarios of disease outbreaks in the less developed countries. These developments in HTS must be matched with continued efforts to improve the current bioinformatics analytic pipelines. Applying SMRT while genome sequencing to investigate bacterial colistin resistance would be made possible to predict resistance mutations, resistance mechanisms, trends, and patterns enabling efficient management of the colistin resistance by healthcare providers and pharmaceutical companies.

## Conclusions

It is known that host, bacterial and environmental factors interact collectively to bring about antibiotic resistance. Therefore, HTS should be applied to a wide range of global collections of bacterial whole genomes to identify and predict new antibiotic drug resistance mutations using appropriate computational and bioinformatics algorithms.

Computational algorithms and tools offer the ability to simulate bacterial genomic mutations while also offering possible clues on the mechanisms that may be shaping these mutations. These can as well be utilized to develop therapeutic interventions that may be used to target both the current and future acquired antibiotic drug resistance mutations.

## Data availability

No data are associated with this article
