# Supplementary material for: Investigating colistin drug resistance: The role of high-throughput sequencing and bioinformatics
Source: F1000Res. 2019 May 20;8:150. Originally published 2019 Feb 4. [Version 2] doi: 10.12688/f1000research.18081.2 (PMC6635981; doi:10.12688/f1000research.18081.2)
Supplement: Peer review history

# Peer review: Reviewer response for version 2

**DOI:** 10.5256/f1000research.21085.r50831

**Published:** 2019-07-23

**Authors:** Enrico Lavezzo

**Affiliations:** 1 Department of Molecular Medicine, University of Padova, Padua, Italy

In this short review, Aruhomukama and co-authors discuss about microbial resistance to antibiotics, in particular to colistin, which seems to be the ‘last resort’ in this era of increasing antibiotic resistance.

 They start with a brief report on colistin resistance prevalence in Africa, then they describe the evolution of colistin resistance from a chromosomal feature to a plasmid-related threat, and finally they discuss about high-throughput sequencing and bioinformatics as essential tools for monitoring and contain the rise of antimicrobial genes.

 The review is very brief, so I understand that it is not possible to enter in too many details. Nonetheless, there are topics that need to be better discussed and explained, especially in the last section about HTS and bioinformatics, otherwise they are, in my opinion, of no help for the reader.

 In the following I list those that are major and minor issues that, to me, the authors should address to improve the quality of the manuscript.

 Major points:

- In the abstract, authors mention the search for novel drug targets as one of the topics included in the review, but I found no further information throughout the article. It should be removed from the abstract.
- In the section “The role of high-throughput sequencing technologies and bioinformatics” there is an extremely vague and imprecise definition of what bioinformatics is. I think this is useless: I suppose that all readers know that there are computer-based algorithms for analyzing genomics data and they would rather benefit from at least a brief description of the main steps that bioinformatics tools perform in applications related to clinical settings. And if there is a gap in the ability of clinicians to use these tools, this should be discussed and the reasons explained, also proposing potential solutions.

- The section “The future direction of HTS” starts with a very general description of the potential provided by HTS to study the microbial world. I suppose the authors are referring to metagenomics, even if this word is never mentioned. I think that this application should be discussed a little bit more extensively together with its role in the clinical setting.

- In the same section, the authors talk about SMRT sequencing platform (PacBio), but the features of this platform are not explained, and no other platforms are discussed for comparative reasons. Why talking about PacBio here? Which is the advantage/disadvantage with respect to other platforms? In this form, this section is completely detached from the rest of the article. 

- Conclusions: the authors state that HTS should be applied for the identification and prediction of drug resistance genes using appropriate bioinformatics tools. But these tools have never been mentioned in the article. Which tools? Do they need to do?

Moreover, they claim that computational algorithms can be applied for developing therapeutic interventions and target drug resistance mutations. Let’s say that this is true, but it is not described in the article so I don’t understand how this can be a conclusion of the paper.

 Minor issues:

- There are some couples of words that are not separated by a white space throughout the text.
  “Withbacteria”, page 4, first columns, line18.
  “phenotypicantibiotic”, page 5, first column, line 5 from the bottom.
- Some sentences need to be rephrased for more clarity:
  “These enzymes have in recent…”, page 4, second columns, line 3.
  “Also, worth noting is…”, page 4, second column, last 3 lines.
  “Furthermore, these have also…”, page 5, second column, line 6.
- “Currently studies” à current studies (page 4, first column, halfway)
- “it is rapidly replacing … in the near future” à it is going to replace?
- “Bioinformatics … usually with the assistance of computer based algorithms” à why usually?

I confirm that I have read this submission and believe that I have an appropriate level of expertise to confirm that it is of an acceptable scientific standard, however I have significant reservations, as outlined above.

# Peer review: Reviewer response for version 2

**DOI:** 10.5256/f1000research.21085.r48746

**Published:** 2019-07-15

**Authors:** Hosam Zowawi, Sharif Hala

**Affiliations:** 1 UQ Centre for Clinical Centre, The University of Queensland, Brisbane, Qld, Australia; 2 King Abdullah University for Science and Technology, Thuwal, Saudi Arabia

We thank the authors for considering our comments and revising their manuscript, their efforts are clearly reflected in this revised version. We wish them all the best and we have no further comments on this article.

We confirm that we have read this submission and believe that we have an appropriate level of expertise to confirm that it is of an acceptable scientific standard.

# Peer review: Reviewer response for version 2

**DOI:** 10.5256/f1000research.21085.r48745

**Published:** 2019-06-19

**Authors:** Alexandre Angers-Loustau

**Affiliations:** 1 Joint Research Centre (JRC), European Commission (EC), Ispra, Italy

I would like to thank the authors for their efforts in addressing my concerns with Version 1.  I have no further comment on this article.

I confirm that I have read this submission and believe that I have an appropriate level of expertise to confirm that it is of an acceptable scientific standard.

# Peer review: Reviewer response for version 1

**DOI:** 10.5256/f1000research.19772.r47772

**Published:** 2019-04-29

**Authors:** Hosam Zowawi, Sharif Hala

**Affiliations:** 1 UQ Centre for Clinical Centre, The University of Queensland, Brisbane, Qld, Australia; 2 King Abdullah University for Science and Technology, Thuwal, Saudi Arabia

The study by Mboowa et al focuses on reviewing the molecular mechanisms underlying the role of Colistin resistance in and the importance of high-throughput sequencing and data analysis in identifying chromosomal and plasmid resistance. The authors explored the spread of colistin resistance genes and gave an example on the spread of mcr-1 in Africa. They gave examples of the lipid A modification to be linked to two component systems. Moreover, they demonstrated the link between other resistance pathways and colistin where they highlighted examples of mobile colistin elements and tetracycline and carbapenem resistance co-transfer and co-exist. Finally, they described the role of High-throughput sequencing technologies and identified several platforms and methods. Overall, the review is interesting and the data presented in the manuscript is somewhat supporting the conclusion. However, there are a few comments that need to be addressed prior to indexing:

**General comments:**

- The manuscript is written in poor English. Several statements are too long and lack clarity, precision, and completeness. The manuscript has to be rewritten in proper English before submitting it for a detailed revision. As an example “The modifications of the lipid A, which reduce and/or abolish the initial charge-based interaction with the polymyxins in bacteria13,15,16 and also the addition of either/or the 4-amino-4- deoxy-L-arabinose (L-Ara4N) and the phosphoethanolamine (PEtn) that ultimately form the basis of colistin resistance in bacteria16, is mediated by chromosomally encoded genes” *Very confusing and needs further explanations.
- Authors need to spell out the abbreviation as commonly used. As an example HTS should be NGS (next generation sequencing) in the manuscript.
- In the introduction section, the authors adapted figure 1 of Africa, which talks about countries are not represented in figure 1 and the figure is not matching what is written. Perhaps a different figure could represent the text better.
- The reference quoted need to be checked as I have noticed addition of unnecessary references or references that do not match the text. Example reference 4, 16, 17 are quoted in some places where it’s not needed have 3 references and do not match the text.
- The abstract ****mentions novel mobile colistin resistance plasmid that was not discussed.

**Specific comments:**

- Examples of bacterial type and specific chromosomal mutations are needed, generalization of the resistance pattern could be misleading especially with chromosomal genes. Also, other known chromosomal genes such as pmrC, crrA/B/C, dedA. Finally, as an example a gene such as *eraR*is known to be linked to colistin only in *Pseudomonas*as a response regulator, therefore, the authors are encouraged to mention that different mutations in different organisms can be identified by NGS.
- Repetition of mcr-1,mcr-2 and mcr-3 sentences several times and not including the rest of the genes all the way to the newly discovered but not published yet mcr-9 and maybe mention the location of such genes.

We confirm that we have read this submission and believe that we have an appropriate level of expertise to confirm that it is of an acceptable scientific standard, however we have significant reservations, as outlined above.

# Response

**Published:** 2019-05-14

**Authors:** Dickson Aruhomukama

**Affiliations:** Makerere University, Uganda

We appreciate all the comments made by the reviewers, these helped us to make the manuscript even much better. In the revised manuscript we addressed the comments made as follows;

- General comments – we totally agree with comment (i) in regards to a number of sections being too long and, lacking clarity, precision, and completeness, we have reviewed the entire manuscript, identified sections with these concerns and have endeavored to make these shorter, more clear, precise and, complete. In regards to comment (ii), we agree that HTS and WGS are interchangeably used, however, since we used HTS in the manuscript we have to choose to maintain the use of HTS. Changes were made to address comment (iii), (iv) and, (v) as well.
- Specific comments – (i) we agree that not all chromosomally mediated resistance mechanisms were discussed, however, we ultimately highlight the potential of WGS in the identification of these as well as acquired resistance mechanisms. Comment (ii) was addressed as well.

# Peer review: Reviewer response for version 1

**DOI:** 10.5256/f1000research.19772.r45068

**Published:** 2019-03-27

**Authors:** Alexandre Angers-Loustau

**Affiliations:** 1 Joint Research Centre (JRC), European Commission (EC), Ispra, Italy

In this review, the authors describe the biology behind the evolution of antimicrobial resistance, with a focus on the resistance against colistin, one of the "last-resort" antibiotics. An alarming development was reported in 2016, where a form of resistance was discovered that could be readily transferred to other bacteria. Researchers first discovered this resistance in China, quickly followed by findings of similar resistance patterns in other countries. These discoveries relied in part on the use of high throughput sequencing.

 The review is very short and it is understandable that, within these four pages, the authors can't expand these themes in too many details. Still, I have a few minor and a few major comments about the current version of the article.

 Minor comments:

- The flow of the different sections of the review is a bit off in my opinion:

          a) The section "Colistin resistance trends in Africa" describes the observed burden of colistin resistance in this continent

           b) the section "Evolution to plasmid mediated colistin resistance …" describes the biology and mechanism of colistin resistance, from chromosomal to plasmid-mediated.

           c) the section "Pan-drug resistance and characteristics…" describes, generally, the phenomena that gives rise to AMR.

 It would make more sense, to me, to rework these sections in the order c), b) then a). 

- The sentence "Furthermore, phenomena such as genome plasticity and pan genomes that have the ability to influence bacterial resistome can only effectively be investigated using HTS and bioinformatics analyses." needs to be better explained and referenced.  Also, pan genomes are not, in my opinion, "phenomena". 
- The section "Evolution to plasmid mediated colistin resistance..." explains the mechanisms of action of chromosome-mediated resistance, then simply lists the genes involved in plasmid-mediated resistance, with no indication about modes of action of these genes, and whether it is different from the ones described previously.
- I noticed at least one incomplete sentence ("Plasmid transfer of the colistin resistance mobile genes, mcr-1, mcr-2, and also mcr-3.") 

Major comments:

- The section on Africa needs to be reworked. First, the Figure says "Adapted from Xavier et al. under a CC-BY 4.0 license". I don't know about the license and rights, but I didn't notice any difference with the original figure, so a more appropriate phrasing would be "Reproduced from…" (or, if I missed it, a better indication on how it was adapted). 
- Second, I don't see how the figure supports the statement "The limited available literature from African settings has reported the mcr-1 gene mediated colistin resistance to be most prevalent in Africa, largely in South Africa, and this covers the largest portion of Africa according to the global map"; according to the figure, detection in Africa does not look more prevalent compared to other continents.
- Finally, the text mentions detection in South Africa, Algeria, Rwanda and Uganda, while the maps shows South Africa, Algeria, Tunisia and Egypt.
- The last section, "The role of high-throughput sequencing …" is very generic, and barely addresses how this applied to colistin resistance, as the tile of the review suggests.  It contain very general statements about the possibilities of HTS for AMR detection, that have been reviewed in more details in a few recent publications. The authors should take the opportunity of assessing how HTS led to the rapid detection of the spread of the newly identified colistin resistance risk, in a way that was not possible before. Relevant studies should include, among others, the following: 

Hasman, H., Hammerum, A.M., Hansen, F., Hendriksen, R.S., Olesen, B., Agersø, Y.,

 Zankari, E., Leekitcharoenphon, P., Stegger, M., Kaas, R.S., et al. (2015). Detection of

*mcr-1 *encoding plasmid-mediated colistin-resistant *Escherichia coli *isolates from human

 bloodstream infection and imported chicken meat ^1^. 

 Kluytmans–van den Bergh, M.F., Huizinga, P., M. J. Bonten, M. Bos, K. De Bruyne, A. W. Friedrich, J. W. Rossen, P. H. Savelkoul, and J. A. Kluytmans. (2009) Presence of mcr-1-positive Enterobacteriaceae in retail chicken meat but not in humans in the Netherlands since 2009 ^2^.

 Yu, C. Y., Ang, G. Y., Chong, T. M., Chin, P. S., Ngeow, Y. F., Yin, W. F., & Chan, K. G. (2016). Complete genome sequencing revealed novel genetic contexts of the mcr-1 gene in Escherichia coli strains ^3^.

I confirm that I have read this submission and believe that I have an appropriate level of expertise to confirm that it is of an acceptable scientific standard, however I have significant reservations, as outlined above.

## References

[1] : Detection of mcr-1 encoding plasmid-mediated colistin-resistant Escherichia coli isolates from human bloodstream infection and imported chicken meat, Denmark 2015. *Euro Surveill*.2015;20(49) : 10.2807/1560-7917.ES.2015.20.49.30085 10.2807/1560-7917.ES.2015.20.49.30085 26676364

[2] : Presence of mcr-1-positive Enterobacteriaceae in retail chicken meat but not in humans in the Netherlands since 2009. *Euro Surveill*.2016;21(9) : 10.2807/1560-7917.ES.2016.21.9.30149 30149 10.2807/1560-7917.ES.2016.21.9.30149 26967540

[3] : Heterogeneous Genetic Location of mcr-1 in Colistin-Resistant Escherichia coli Isolates from Humans and Retail Chicken Meat in Switzerland: Emergence of mcr-1-Carrying IncK2 Plasmids. *Antimicrob Agents Chemother*.61(11) : 10.1128/AAC.01245-17 10.1128/AAC.01245-17 28848010PMC5655086

# Response

**Published:** 2019-05-14

**Authors:** Dickson Aruhomukama

**Affiliations:** Makerere University, Uganda

We appreciate all the comments made by the reviewer, these helped us to make the manuscript even much better. In the revised manuscript we addressed the comments made as follows;

- Minor comments – (i) we respect the opinion of the reviewer in regards to reworking the sections, however, we noted that this would grossly distort the manuscript and hence choose to maintain the order as is. We agree with the concerns raised in comments (ii), (iii) and, (iv), we addressed each of these as reflected in version 2 of the manuscript.  

- Major comments - we agree with the reviewer's comments in regards reworking the section on Africa (i.e. comments i, ii and, iii), these have all been addressed in version 2 of the manuscript. In regards to comment (iv), we reviewed the articles suggested by the reviewer and added a section that briefly describes how HTS leads to the rapid detection of the spread of the newly identified colistin resistance risk. The articles were referenced as well.   
